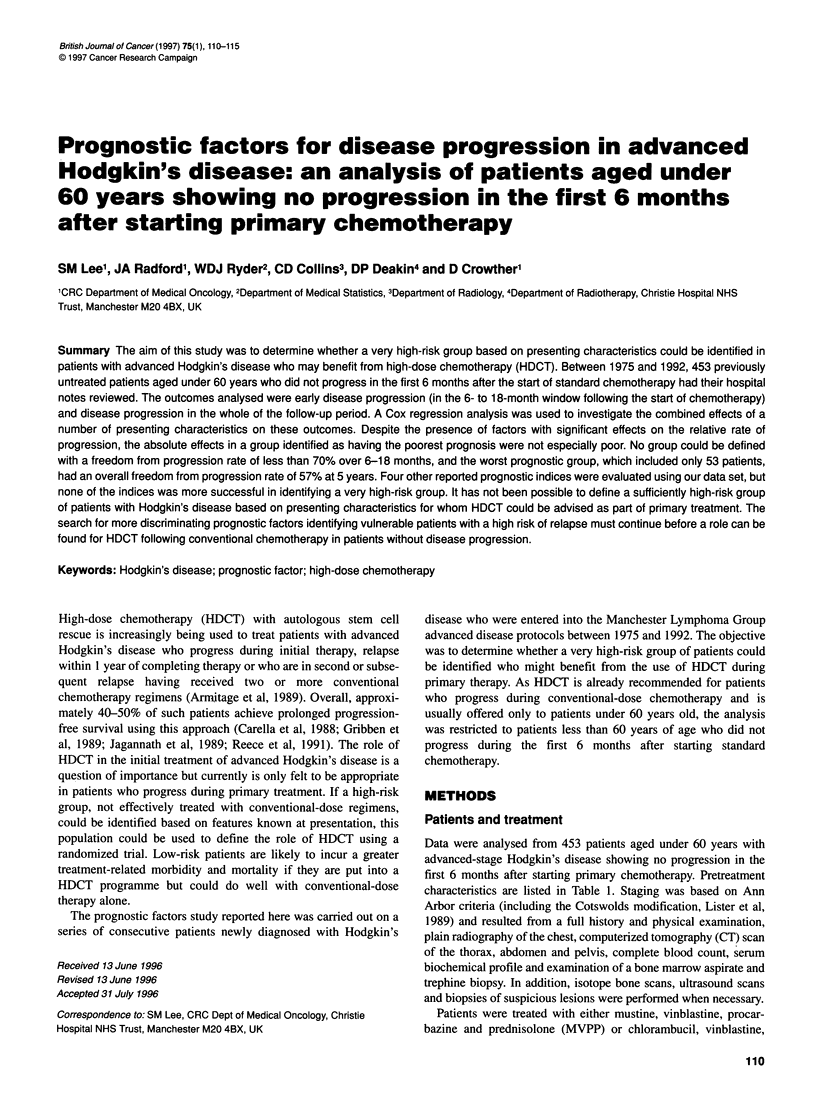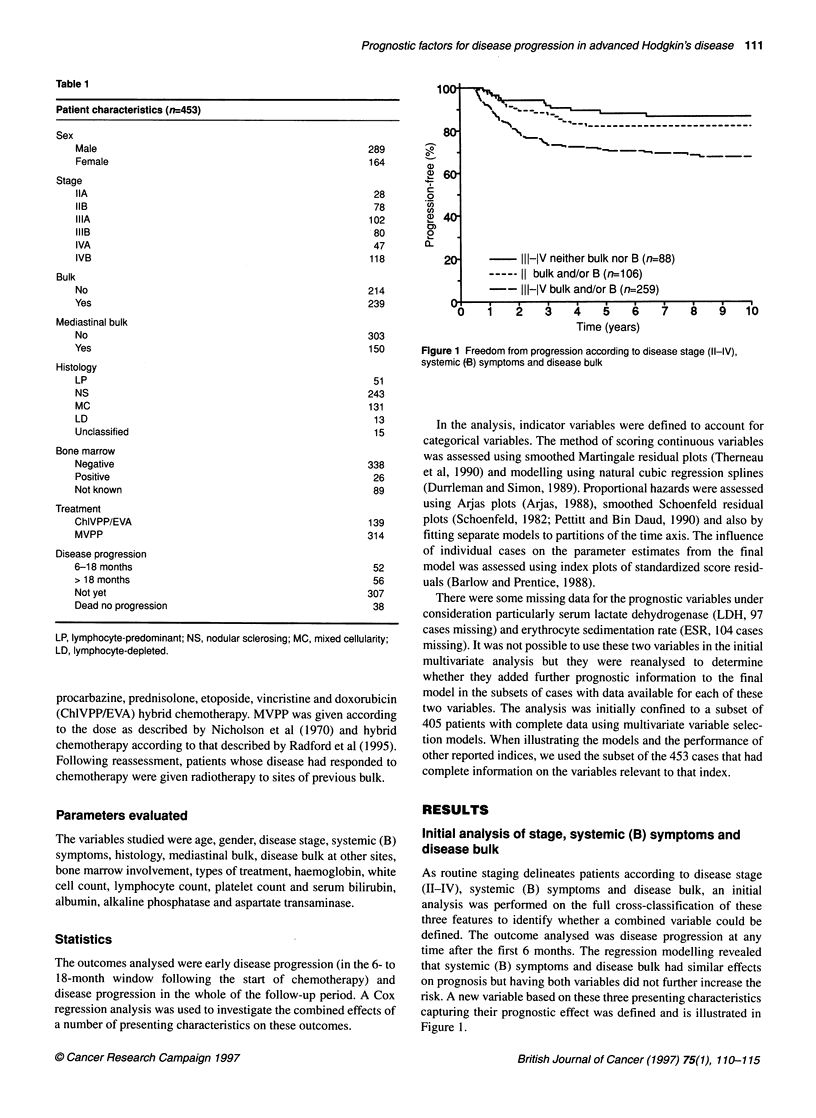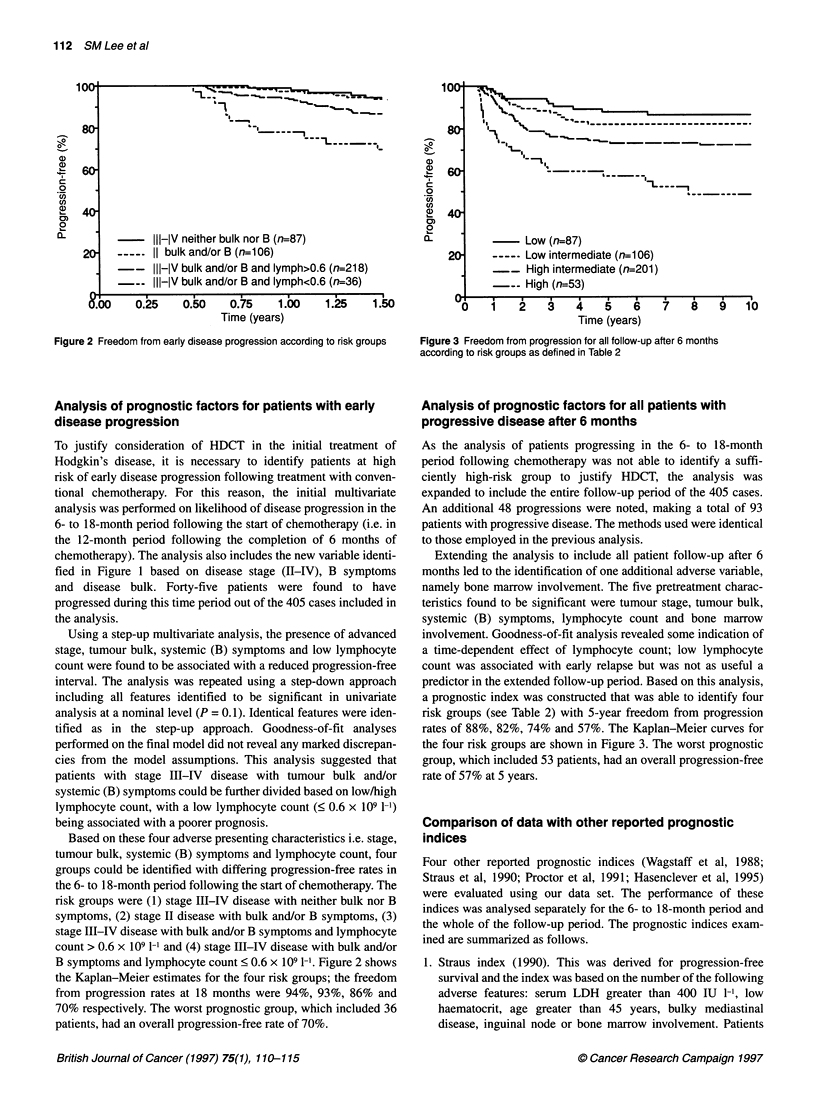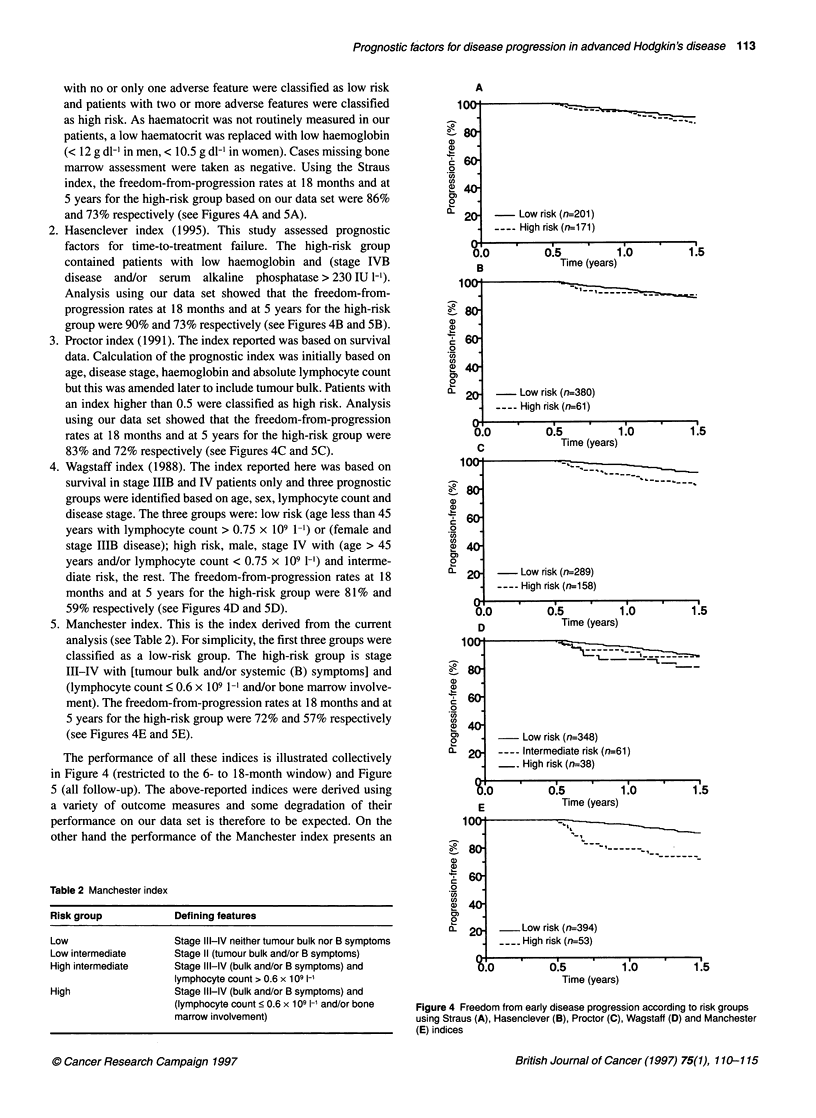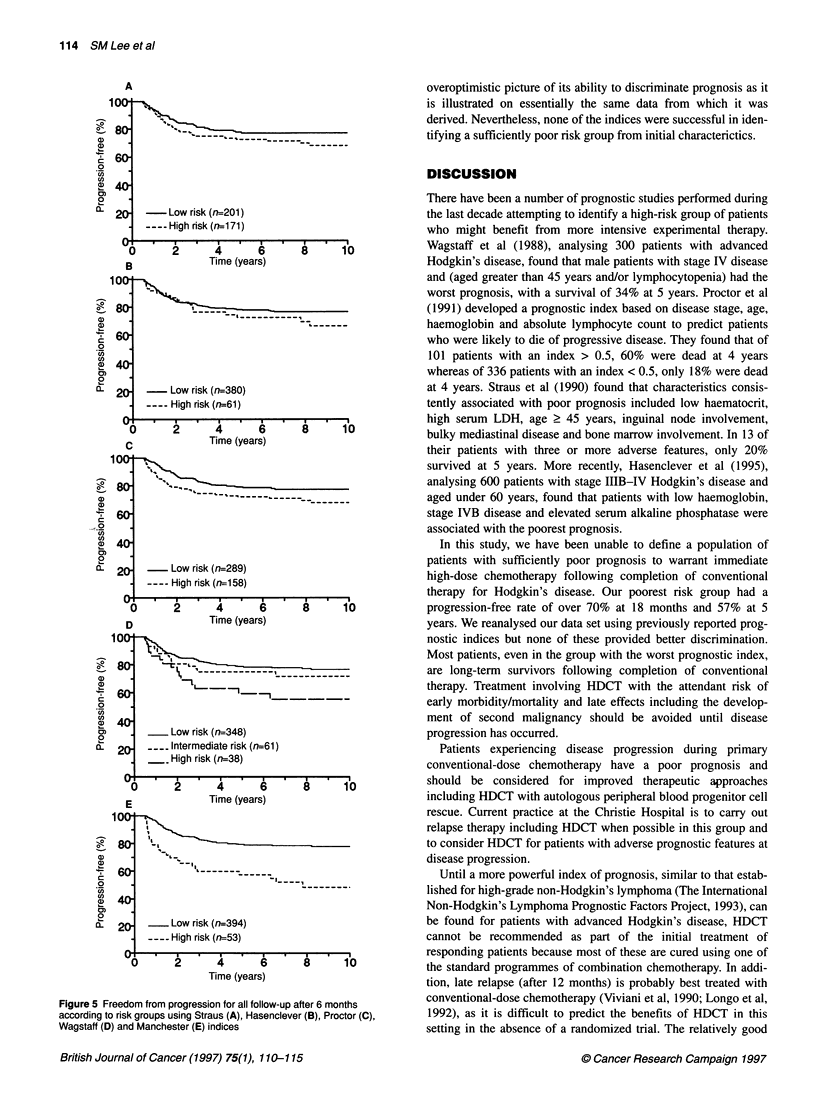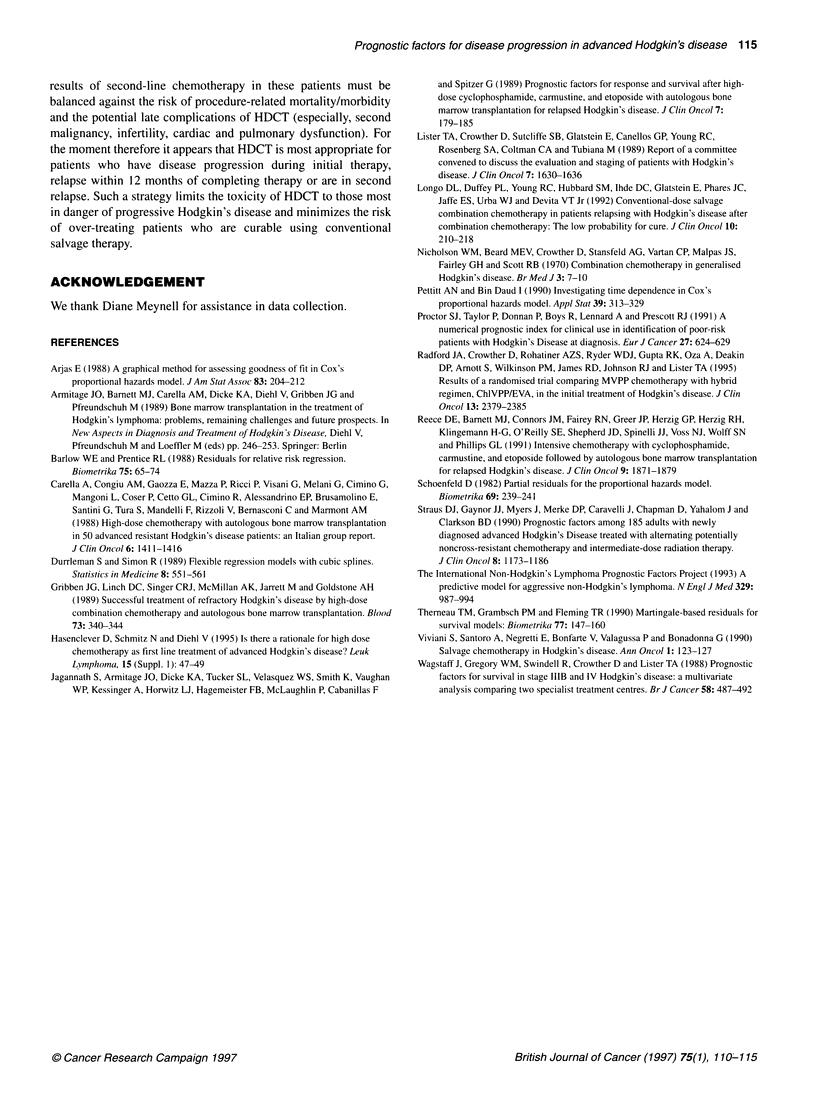# Prognostic factors for disease progression in advanced Hodgkin's disease: an analysis of patients aged under 60 years showing no progression in the first 6 months after starting primary chemotherapy.

**DOI:** 10.1038/bjc.1997.18

**Published:** 1997

**Authors:** S. M. Lee, J. A. Radford, W. D. Ryder, C. D. Collins, D. P. Deakin, D. Crowther

**Affiliations:** CRC Department of Medical Oncology, Christie Hospital NHS Trust, Manchester, UK.

## Abstract

The aim of this study was to determine whether a very high-risk group based on presenting characteristics could be identified in patients with advanced Hodgkin's disease who may benefit from high-dose chemotherapy (HDCT). Between 1975 and 1992, 453 previously untreated patients aged under 60 years who did not progress in the first 6 months after the start of standard chemotherapy had their hospital notes reviewed. The outcomes analysed were early disease progression (in the 6- to 18-month window following the start of chemotherapy) and disease progression in the whole of the follow-up period. A Cox regression analysis was used to investigate the combined effects of a number of presenting characteristics on these outcomes. Despite the presence of factors with significant effects on the relative rate of progression, the absolute effects in a group identified as having the poorest prognosis were not especially poor. No group could be defined with a freedom from progression rate of less than 70% over 6-18 months, and the worst prognostic group, which included only 53 patients, had an overall freedom from progression rate of 57% at 5 years. Four other reported prognostic indices were evaluated using our data set, but none of the indices was more successful in identifying a very high-risk group. It has not been possible to define a sufficiently high-risk group of patients with Hodgkin's disease based on presenting characteristics for whom HDCT could be advised as part of primary treatment. The search for more discriminating prognostic factors identifying vulnerable patients with a high risk of relapse must continue before a role can be found for HDCT following conventional chemotherapy in patients without disease progression.


British Journal of Cancer (1997) 75(1), 110–115
© 1997 Cancer Research Campaign

### Prognostic factors for disease progression in advanced Hodgkin's disease: an analysis of patients aged under 60 years showing no progression in the first 6 months after starting primary chemotherapy

**SM Lee[1], JA Radford[1], WDJ Ryder[2], CD Collins[3], DP Deakin[4] and D Crowther[1]**

[1]CRC Department of Medical Oncology, [2]Department of Medical Statistics, [3]Department of Radiology, [4]Department of Radiotherapy, Christie Hospital NHS Trust, Manchester M20 4BX, UK

**Summary** The aim of this study was to determine whether a very high-risk group based on presenting characteristics could be identified in patients with advanced Hodgkin's disease who may benefit from high-dose chemotherapy (HDCT). Between 1975 and 1992, 453 previously untreated patients aged under 60 years who did not progress in the first 6 months after the start of standard chemotherapy had their hospital notes reviewed. The outcomes analysed were early disease progression (in the 6- to 18-month window following the start of chemotherapy) and disease progression in the whole of the follow-up period. A Cox regression analysis was used to investigate the combined effects of a number of presenting characteristics on these outcomes. Despite the presence of factors with significant effects on the relative rate of progression, the absolute effects in a group identified as having the poorest prognosis were not especially poor. No group could be defined with a freedom from progression rate of less than 70% over 6–18 months, and the worst prognostic group, which included only 53 patients, had an overall freedom from progression rate of 57% at 5 years. Four other reported prognostic indices were evaluated using our data set, but none of the indices was more successful in identifying a very high-risk group. It has not been possible to define a sufficiently high-risk group of patients with Hodgkin's disease based on presenting characteristics for whom HDCT could be advised as part of primary treatment. The search for more discriminating prognostic factors identifying vulnerable patients with a high risk of relapse must continue before a role can be found for HDCT following conventional chemotherapy in patients without disease progression.

**Keywords:** Hodgkin's disease; prognostic factor; high-dose chemotherapy

High-dose chemotherapy (HDCT) with autologous stem cell rescue is increasingly being used to treat patients with advanced Hodgkin's disease who progress during initial therapy, relapse within 1 year of completing therapy or who are in second or subsequent relapse having received two or more conventional chemotherapy regimens (Armitage et al, 1989). Overall, approximately 40–50% of such patients achieve prolonged progression-free survival using this approach (Carella et al, 1988; Gribben et al, 1989; Jagannath et al, 1989; Reece et al, 1991). The role of HDCT in the initial treatment of advanced Hodgkin's disease is a question of importance but currently is only felt to be appropriate in patients who progress during primary treatment. If a high-risk group, not effectively treated with conventional-dose regimens, could be identified based on features known at presentation, this population could be used to define the role of HDCT using a randomized trial. Low-risk patients are likely to incur a greater treatment-related morbidity and mortality if they are put into a HDCT programme but could do well with conventional-dose therapy alone.

The prognostic factors study reported here was carried out on a series of consecutive patients newly diagnosed with Hodgkin's disease who were entered into the Manchester Lymphoma Group advanced disease protocols between 1975 and 1992. The objective was to determine whether a very high-risk group of patients could be identified who might benefit from the use of HDCT during primary therapy. As HDCT is already recommended for patients who progress during conventional-dose chemotherapy and is usually offered only to patients under 60 years old, the analysis was restricted to patients less than 60 years of age who did not progress during the first 6 months after starting standard chemotherapy.

*Received 13 June 1996*
*Revised 13 June 1996*
*Accepted 31 July 1996*

*Correspondence to:* SM Lee, CRC Dept of Medical Oncology, Christie Hospital NHS Trust, Manchester M20 4BX, UK

### METHODS

### Patients and treatment

Data were analysed from 453 patients aged under 60 years with advanced-stage Hodgkin's disease showing no progression in the first 6 months after starting primary chemotherapy. Pretreatment characteristics are listed in Table 1. Staging was based on Ann Arbor criteria (including the Cotswolds modification, Lister et al, 1989) and resulted from a full history and physical examination, plain radiography of the chest, computerized tomography (CT) scan of the thorax, abdomen and pelvis, complete blood count, serum biochemical profile and examination of a bone marrow aspirate and trephine biopsy. In addition, isotope bone scans, ultrasound scans and biopsies of suspicious lesions were performed when necessary.

Patients were treated with either mustine, vinblastine, procarbazine and prednisolone (MVPP) or chlorambucil, vinblastine,

**Table 1**

| Patient characteristics ($n$=453) | |
|---|---|
| Sex | |
| Male | 289 |
| Female | 164 |
| Stage | |
| IIA | 28 |
| IIB | 78 |
| IIIA | 102 |
| IIIB | 80 |
| IVA | 47 |
| IVB | 118 |
| Bulk | |
| No | 214 |
| Yes | 239 |
| Mediastinal bulk | |
| No | 303 |
| Yes | 150 |
| Histology | |
| LP | 51 |
| NS | 243 |
| MC | 131 |
| LD | 13 |
| Unclassified | 15 |
| Bone marrow | |
| Negative | 338 |
| Positive | 26 |
| Not known | 89 |
| Treatment | |
| ChlVPP/EVA | 139 |
| MVPP | 314 |
| Disease progression | |
| 6–18 months | 52 |
| > 18 months | 56 |
| Not yet | 307 |
| Dead no progression | 38 |

LP, lymphocyte-predominant; NS, nodular sclerosing; MC, mixed cellularity; LD, lymphocyte-depleted.

procarbazine, prednisolone, etoposide, vincristine and doxorubicin (ChlVPP/EVA) hybrid chemotherapy. MVPP was given according to the dose as described by Nicholson et al (1970) and hybrid chemotherapy according to that described by Radford et al (1995). Following reassessment, patients whose disease had responded to chemotherapy were given radiotherapy to sites of previous bulk.

### Parameters evaluated

The variables studied were age, gender, disease stage, systemic (B) symptoms, histology, mediastinal bulk, disease bulk at other sites, bone marrow involvement, types of treatment, haemoglobin, white cell count, lymphocyte count, platelet count and serum bilirubin, albumin, alkaline phosphatase and aspartate transaminase.

### Statistics

The outcomes analysed were early disease progression (in the 6- to 18-month window following the start of chemotherapy) and disease progression in the whole of the follow-up period. A Cox regression analysis was used to investigate the combined effects of a number of presenting characteristics on these outcomes.

**Figure 1** Freedom from progression according to disease stage (II–IV), systemic (B) symptoms and disease bulk

In the analysis, indicator variables were defined to account for categorical variables. The method of scoring continuous variables was assessed using smoothed Martingale residual plots (Therneau et al, 1990) and modelling using natural cubic regression splines (Durrleman and Simon, 1989). Proportional hazards were assessed using Arjas plots (Arjas, 1988), smoothed Schoenfeld residual plots (Schoenfeld, 1982; Pettitt and Bin Daud, 1990) and also by fitting separate models to partitions of the time axis. The influence of individual cases on the parameter estimates from the final model was assessed using index plots of standardized score residuals (Barlow and Prentice, 1988).

There were some missing data for the prognostic variables under consideration particularly serum lactate dehydrogenase (LDH, 97 cases missing) and erythrocyte sedimentation rate (ESR, 104 cases missing). It was not possible to use these two variables in the initial multivariate analysis but they were reanalysed to determine whether they added further prognostic information to the final model in the subsets of cases with data available for each of these two variables. The analysis was initially confined to a subset of 405 patients with complete data using multivariate variable selection models. When illustrating the models and the performance of other reported indices, we used the subset of the 453 cases that had complete information on the variables relevant to that index.

### RESULTS

### Initial analysis of stage, systemic (B) symptoms and disease bulk

As routine staging delineates patients according to disease stage (II–IV), systemic (B) symptoms and disease bulk, an initial analysis was performed on the full cross-classification of these three features to identify whether a combined variable could be defined. The outcome analysed was disease progression at any time after the first 6 months. The regression modelling revealed that systemic (B) symptoms and disease bulk had similar effects on prognosis but having both variables did not further increase the risk. A new variable based on these three presenting characteristics capturing their prognostic effect was defined and is illustrated in Figure 1.

© Cancer Research Campaign 1997

**Figure 2** Freedom from early disease progression according to risk groups

**Figure 3** Freedom from progression for all follow-up after 6 months according to risk groups as defined in Table 2

### Analysis of prognostic factors for patients with early disease progression

To justify consideration of HDCT in the initial treatment of Hodgkin's disease, it is necessary to identify patients at high risk of early disease progression following treatment with conventional chemotherapy. For this reason, the initial multivariate analysis was performed on likelihood of disease progression in the 6- to 18-month period following the start of chemotherapy (i.e. in the 12-month period following the completion of 6 months of chemotherapy). The analysis also includes the new variable identified in Figure 1 based on disease stage (II–IV), B symptoms and disease bulk. Forty-five patients were found to have progressed during this time period out of the 405 cases included in the analysis.

Using a step-up multivariate analysis, the presence of advanced stage, tumour bulk, systemic (B) symptoms and low lymphocyte count were found to be associated with a reduced progression-free interval. The analysis was repeated using a step-down approach including all features identified to be significant in univariate analysis at a nominal level ($P = 0.1$). Identical features were identified as in the step-up approach. Goodness-of-fit analyses performed on the final model did not reveal any marked discrepancies from the model assumptions. This analysis suggested that patients with stage III–IV disease with tumour bulk and/or systemic (B) symptoms could be further divided based on low/high lymphocyte count, with a low lymphocyte count ($\leq 0.6 \times 10^9$ $l^{-1}$) being associated with a poorer prognosis.

Based on these four adverse presenting characteristics i.e. stage, tumour bulk, systemic (B) symptoms and lymphocyte count, four groups could be identified with differing progression-free rates in the 6- to 18-month period following the start of chemotherapy. The risk groups were (1) stage III–IV disease with neither bulk nor B symptoms, (2) stage II disease with bulk and/or B symptoms, (3) stage III–IV disease with bulk and/or B symptoms and lymphocyte count $> 0.6 \times 10^9$ $l^{-1}$ and (4) stage III–IV disease with bulk and/or B symptoms and lymphocyte count $\leq 0.6 \times 10^9$ $l^{-1}$. Figure 2 shows the Kaplan–Meier estimates for the four risk groups; the freedom from progression rates at 18 months were 94%, 93%, 86% and 70% respectively. The worst prognostic group, which included 36 patients, had an overall progression-free rate of 70%.

### Analysis of prognostic factors for all patients with progressive disease after 6 months

As the analysis of patients progressing in the 6- to 18-month period following chemotherapy was not able to identify a sufficiently high-risk group to justify HDCT, the analysis was expanded to include the entire follow-up period of the 405 cases. An additional 48 progressions were noted, making a total of 93 patients with progressive disease. The methods used were identical to those employed in the previous analysis.

Extending the analysis to include all patient follow-up after 6 months led to the identification of one additional adverse variable, namely bone marrow involvement. The five pretreatment characteristics found to be significant were tumour stage, tumour bulk, systemic (B) symptoms, lymphocyte count and bone marrow involvement. Goodness-of-fit analysis revealed some indication of a time-dependent effect of lymphocyte count; low lymphocyte count was associated with early relapse but was not as useful a predictor in the extended follow-up period. Based on this analysis, a prognostic index was constructed that was able to identify four risk groups (see Table 2) with 5-year freedom from progression rates of 88%, 82%, 74% and 57%. The Kaplan–Meier curves for the four risk groups are shown in Figure 3. The worst prognostic group, which included 53 patients, had an overall progression-free rate of 57% at 5 years.

### Comparison of data with other reported prognostic indices

Four other reported prognostic indices (Wagstaff et al, 1988; Straus et al, 1990; Proctor et al, 1991; Hasenclever et al, 1995) were evaluated using our data set. The performance of these indices was analysed separately for the 6- to 18-month period and the whole of the follow-up period. The prognostic indices examined are summarized as follows.

1. Straus index (1990). This was derived for progression-free survival and the index was based on the number of the following adverse features: serum LDH greater than 400 IU $l^{-1}$, low haematocrit, age greater than 45 years, bulky mediastinal disease, inguinal node or bone marrow involvement. Patients

© Cancer Research Campaign 1997

with no or only one adverse feature were classified as low risk and patients with two or more adverse features were classified as high risk. As haematocrit was not routinely measured in our patients, a low haematocrit was replaced with low haemoglobin ($< 12$ g $dl^{-1}$ in men, $< 10.5$ g $dl^{-1}$ in women). Cases missing bone marrow assessment were taken as negative. Using the Straus index, the freedom-from-progression rates at 18 months and at 5 years for the high-risk group based on our data set were 86% and 73% respectively (see Figures 4A and 5A).

2. Hasenclever index (1995). This study assessed prognostic factors for time-to-treatment failure. The high-risk group contained patients with low haemoglobin and (stage IVB disease and/or serum alkaline phosphatase $> 230$ IU $l^{-1}$). Analysis using our data set showed that the freedom-from-progression rates at 18 months and at 5 years for the high-risk group were 90% and 73% respectively (see Figures 4B and 5B).
3. Proctor index (1991). The index reported was based on survival data. Calculation of the prognostic index was initially based on age, disease stage, haemoglobin and absolute lymphocyte count but this was amended later to include tumour bulk. Patients with an index higher than 0.5 were classified as high risk. Analysis using our data set showed that the freedom-from-progression rates at 18 months and at 5 years for the high-risk group were 83% and 72% respectively (see Figures 4C and 5C).
4. Wagstaff index (1988). The index reported here was based on survival in stage IIIB and IV patients only and three prognostic groups were identified based on age, sex, lymphocyte count and disease stage. The three groups were: low risk (age less than 45 years with lymphocyte count $> 0.75 \times 10^9$ $l^{-1}$) or (female and stage IIIB disease); high risk, male, stage IV with (age $> 45$ years and/or lymphocyte count $< 0.75 \times 10^9$ $l^{-1}$) and intermediate risk, the rest. The freedom-from-progression rates at 18 months and at 5 years for the high-risk group were 81% and 59% respectively (see Figures 4D and 5D).
5. Manchester index. This is the index derived from the current analysis (see Table 2). For simplicity, the first three groups were classified as a low-risk group. The high-risk group is stage III–IV with [tumour bulk and/or systemic (B) symptoms] and (lymphocyte count $\leq 0.6 \times 10^9$ $l^{-1}$ and/or bone marrow involvement). The freedom-from-progression rates at 18 months and at 5 years for the high-risk group were 72% and 57% respectively (see Figures 4E and 5E).

The performance of all these indices is illustrated collectively in Figure 4 (restricted to the 6- to 18-month window) and Figure 5 (all follow-up). The above-reported indices were derived using a variety of outcome measures and some degradation of their performance on our data set is therefore to be expected. On the other hand the performance of the Manchester index presents an

**Table 2** Manchester index

| Risk group | Defining features |
|---|---|
| Low | Stage III–IV neither tumour bulk nor B symptoms |
| Low intermediate | Stage II (tumour bulk and/or B symptoms) |
| High intermediate | Stage III–IV (bulk and/or B symptoms) and lymphocyte count $> 0.6 \times 10^9$ $l^{-1}$ |
| High | Stage III–IV (bulk and/or B symptoms) and (lymphocyte count $\leq 0.6 \times 10^9$ $l^{-1}$ and/or bone marrow involvement) |

**Figure 4** Freedom from early disease progression according to risk groups using Straus (**A**), Hasenclever (**B**), Proctor (**C**), Wagstaff (**D**) and Manchester (**E**) indices

*© Cancer Research Campaign 1997*

**Figure 5** Freedom from progression for all follow-up after 6 months according to risk groups using Straus (**A**), Hasenclever (**B**), Proctor (**C**), Wagstaff (**D**) and Manchester (**E**) indices overoptimistic picture of its ability to discriminate prognosis as it is illustrated on essentially the same data from which it was derived. Nevertheless, none of the indices were successful in identifying a sufficiently poor risk group from initial characterictics.

### DISCUSSION

There have been a number of prognostic studies performed during the last decade attempting to identify a high-risk group of patients who might benefit from more intensive experimental therapy. Wagstaff et al (1988), analysing 300 patients with advanced Hodgkin's disease, found that male patients with stage IV disease and (aged greater than 45 years and/or lymphocytopenia) had the worst prognosis, with a survival of 34% at 5 years. Proctor et al (1991) developed a prognostic index based on disease stage, age, haemoglobin and absolute lymphocyte count to predict patients who were likely to die of progressive disease. They found that of 101 patients with an index > 0.5, 60% were dead at 4 years whereas of 336 patients with an index < 0.5, only 18% were dead at 4 years. Straus et al (1990) found that characteristics consistently associated with poor prognosis included low haematocrit, high serum LDH, age ≥ 45 years, inguinal node involvement, bulky mediastinal disease and bone marrow involvement. In 13 of their patients with three or more adverse features, only 20% survived at 5 years. More recently, Hasenclever et al (1995), analysing 600 patients with stage IIIB–IV Hodgkin's disease and aged under 60 years, found that patients with low haemoglobin, stage IVB disease and elevated serum alkaline phosphatase were associated with the poorest prognosis.

In this study, we have been unable to define a population of patients with sufficiently poor prognosis to warrant immediate high-dose chemotherapy following completion of conventional therapy for Hodgkin's disease. Our poorest risk group had a progression-free rate of over 70% at 18 months and 57% at 5 years. We reanalysed our data set using previously reported prognostic indices but none of these provided better discrimination. Most patients, even in the group with the worst prognostic index, are long-term survivors following completion of conventional therapy. Treatment involving HDCT with the attendant risk of early morbidity/mortality and late effects including the development of second malignancy should be avoided until disease progression has occurred.

Patients experiencing disease progression during primary conventional-dose chemotherapy have a poor prognosis and should be considered for improved therapeutic approaches including HDCT with autologous peripheral blood progenitor cell rescue. Current practice at the Christie Hospital is to carry out relapse therapy including HDCT when possible in this group and to consider HDCT for patients with adverse prognostic features at disease progression.

Until a more powerful index of prognosis, similar to that established for high-grade non-Hodgkin's lymphoma (The International Non-Hodgkin's Lymphoma Prognostic Factors Project, 1993), can be found for patients with advanced Hodgkin's disease, HDCT cannot be recommended as part of the initial treatment of responding patients because most of these are cured using one of the standard programmes of combination chemotherapy. In addition, late relapse (after 12 months) is probably best treated with conventional-dose chemotherapy (Viviani et al, 1990; Longo et al, 1992), as it is difficult to predict the benefits of HDCT in this setting in the absence of a randomized trial. The relatively good

© Cancer Research Campaign 1997

results of second-line chemotherapy in these patients must be balanced against the risk of procedure-related mortality/morbidity and the potential late complications of HDCT (especially, second malignancy, infertility, cardiac and pulmonary dysfunction). For the moment therefore it appears that HDCT is most appropriate for patients who have disease progression during initial therapy, relapse within 12 months of completing therapy or are in second relapse. Such a strategy limits the toxicity of HDCT to those most in danger of progressive Hodgkin's disease and minimizes the risk of over-treating patients who are curable using conventional salvage therapy.

### ACKNOWLEDGEMENT

We thank Diane Meynell for assistance in data collection.

### REFERENCES

Arjas E (1988) A graphical method for assessing goodness of fit in Cox's proportional hazards model. *J Am Stat Assoc* **83:** 204–212

Armitage JO, Barnett MJ, Carella AM, Dicke KA, Diehl V, Gribben JG and Pfreundschuh M (1989) Bone marrow transplantation in the treatment of Hodgkin's lymphoma: problems, remaining challenges and future prospects. In *New Aspects in Diagnosis and Treatment of Hodgkin's Disease,* Diehl V, Pfreundschuh M and Loeffler M (eds) pp. 246–253. Springer: Berlin

Barlow WE and Prentice RL (1988) Residuals for relative risk regression. *Biometrika* **75:** 65–74

Carella A, Congiu AM, Gaozza E, Mazza P, Ricci P, Visani G, Melani G, Cimino G, Mangoni L, Coser P, Cetto GL, Cimino R, Alessandrino EP, Brusamolino E, Santini G, Tura S, Mandelli F, Rizzoli V, Bernasconi C and Marmont AM (1988) High-dose chemotherapy with autologous bone marrow transplantation in 50 advanced resistant Hodgkin's disease patients: an Italian group report. *J Clin Oncol* **6:** 1411–1416

Durrleman S and Simon R (1989) Flexible regression models with cubic splines. *Statistics in Medicine* **8:** 551–561

Gribben JG, Linch DC, Singer CRJ, McMillan AK, Jarrett M and Goldstone AH (1989) Successful treatment of refractory Hodgkin's disease by high-dose combination chemotherapy and autologous bone marrow transplantation. *Blood* **73:** 340–344

Hasenclever D, Schmitz N and Diehl V (1995) Is there a rationale for high dose chemotherapy as first line treatment of advanced Hodgkin's disease? *Leuk Lymphoma,* **15** (Suppl. 1): 47–49

Jagannath S, Armitage JO, Dicke KA, Tucker SL, Velasquez WS, Smith K, Vaughan WP, Kessinger A, Horwitz LJ, Hagemeister FB, McLaughlin P, Cabanillas F and Spitzer G (1989) Prognostic factors for response and survival after high-dose cyclophosphamide, carmustine, and etoposide with autologous bone marrow transplantation for relapsed Hodgkin's disease. *J Clin Oncol* **7:** 179–185

Lister TA, Crowther D, Sutcliffe SB, Glatstein E, Canellos GP, Young RC, Rosenberg SA, Coltman CA and Tubiana M (1989) Report of a committee convened to discuss the evaluation and staging of patients with Hodgkin's disease. *J Clin Oncol* **7:** 1630–1636

Longo DL, Duffey PL, Young RC, Hubbard SM, Ihde DC, Glatstein E, Phares JC, Jaffe ES, Urba WJ and Devita VT Jr (1992) Conventional-dose salvage combination chemotherapy in patients relapsing with Hodgkin's disease after combination chemotherapy: The low probability for cure. *J Clin Oncol* **10:** 210–218

Nicholson WM, Beard MEV, Crowther D, Stansfeld AG, Vartan CP, Malpas JS, Fairley GH and Scott RB (1970) Combination chemotherapy in generalised Hodgkin's disease. *Br Med J* **3:** 7–10

Pettitt AN and Bin Daud I (1990) Investigating time dependence in Cox's proportional hazards model. *Appl Stat* **39:** 313–329

Proctor SJ, Taylor P, Donnan P, Boys R, Lennard A and Prescott RJ (1991) A numerical prognostic index for clinical use in identification of poor-risk patients with Hodgkin's Disease at diagnosis. *Eur J Cancer* **27:** 624–629

Radford JA, Crowther D, Rohatiner AZS, Ryder WDJ, Gupta RK, Oza A, Deakin DP, Arnott S, Wilkinson PM, James RD, Johnson RJ and Lister TA (1995) Results of a randomised trial comparing MVPP chemotherapy with hybrid regimen, ChlVPP/EVA, in the initial treatment of Hodgkin's disease. *J Clin Oncol* **13:** 2379–2385

Reece DE, Barnett MJ, Connors JM, Fairey RN, Greer JP, Herzig GP, Herzig RH, Klingemann H-G, O'Reilly SE, Shepherd JD, Spinelli JJ, Voss NJ, Wolff SN and Phillips GL (1991) Intensive chemotherapy with cyclophosphamide, carmustine, and etoposide followed by autologous bone marrow transplantation for relapsed Hodgkin's disease. *J Clin Oncol* **9:** 1871–1879

Schoenfeld D (1982) Partial residuals for the proportional hazards model. *Biometrika* **69:** 239–241

Straus DJ, Gaynor JJ, Myers J, Merke DP, Caravelli J, Chapman D, Yahalom J and Clarkson BD (1990) Prognostic factors among 185 adults with newly diagnosed advanced Hodgkin's Disease treated with alternating potentially noncross-resistant chemotherapy and intermediate-dose radiation therapy. *J Clin Oncol* **8:** 1173–1186

The International Non-Hodgkin's Lymphoma Prognostic Factors Project (1993) A predictive model for aggressive non-Hodgkin's lymphoma. *N Engl J Med* **329:** 987–994

Therneau TM, Grambsch PM and Fleming TR (1990) Martingale-based residuals for survival models: *Biometrika* **77:** 147–160

Viviani S, Santoro A, Negretti E, Bonfarte V, Valagussa P and Bonadonna G (1990) Salvage chemotherapy in Hodgkin's disease. *Ann Oncol* **1:** 123–127

Wagstaff J, Gregory WM, Swindell R, Crowther D and Lister TA (1988) Prognostic factors for survival in stage IIIB and IV Hodgkin's disease: a multivariate analysis comparing two specialist treatment centres. *Br J Cancer* **58:** 487–492

© Cancer Research Campaign 1997